# Supplementary material for: Extracellular LGALS3BP regulates neural progenitor position and relates to human cortical complexity
Source: Nat Commun. 2021 Nov 2;12:6298. doi: 10.1038/s41467-021-26447-w (PMC8564519; doi:10.1038/s41467-021-26447-w)
Supplement: Supplementary file 1 — Supplementary information. [file 41467_2021_26447_MOESM1_ESM.pdf]

Supplementary Figures

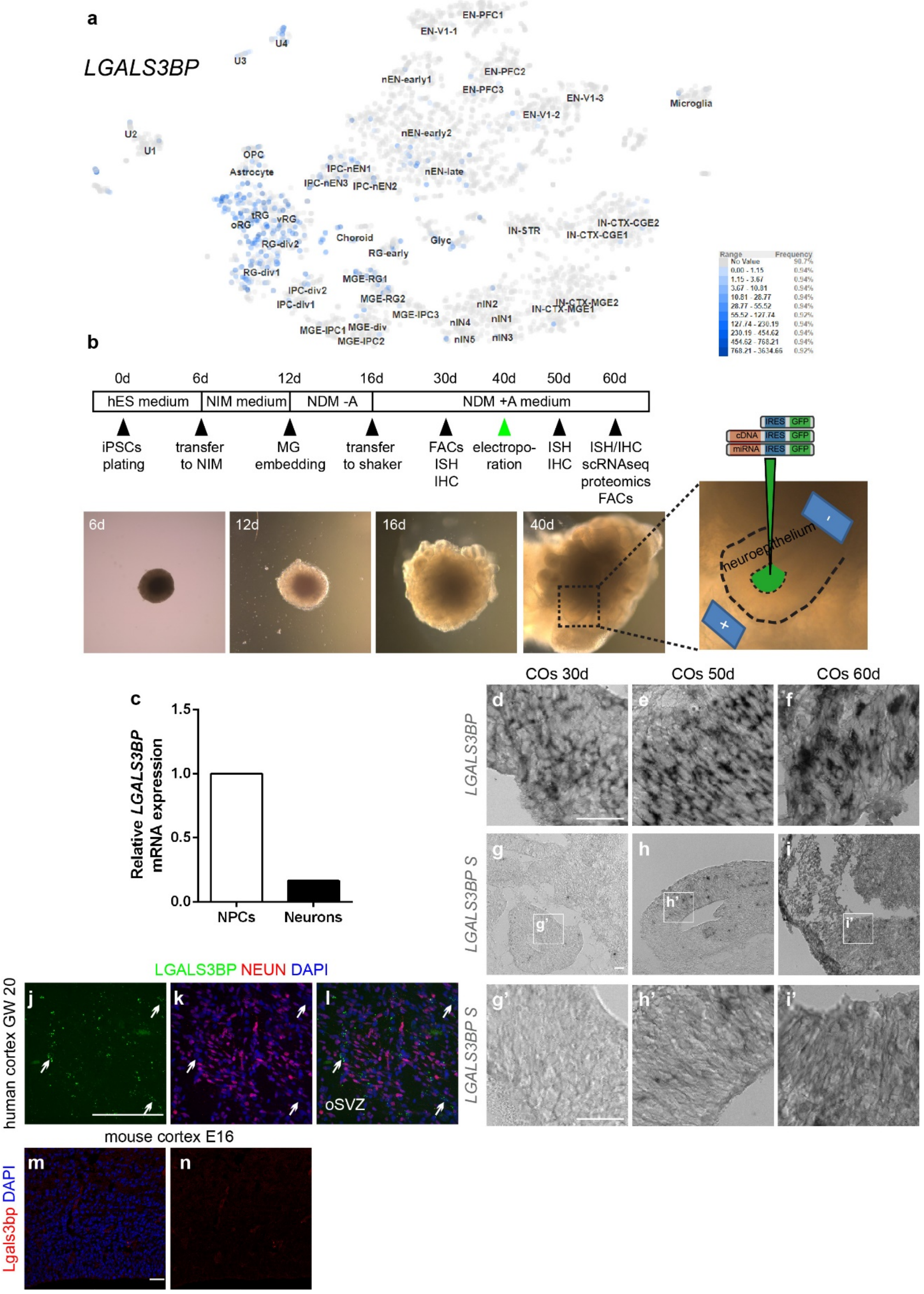

Supplementary Figure 1 Description. Expression of LGALS3BP in human progenitors and neurons (related to Figure 1). a, tSNE plots colored by gene expression in 4,261 single cells isolated from human embryonic cortical tissues[1]. The expression of LGALS3BP is presented in a color-code fashion showing the range of the expression levels of the mRNA and the frequency of the cells exhibiting each expression range. **b,** Schematic representation of the main experimental procedures relative to COs used in the present study. **c,** Quantification of mRNA levels of *LGALS3BP* in 2D neural progenitors and neurons 20 days post differentiation by qPCR shows the reduction of *LGALS3BP* expression upon neuronal induction. Data are shown as fold change over the NPCs as mean, batches=1, n=2. **d-f,** *In situ* hybridization depicting mRNA expression of *LGALS3BP* in 30d, 50d and 60d control COs (higher magnification of the pictures depicted in Fig.1), batches=1, organoids=3, ventricles=3. **g-i',** *In situ* hybridization using a sense RNA probe against *LGALS3BP* in 30d, 50d and 60d control COs. **j-n,** Micrograph of human fetal (j-l) and mouse (m,n) cortical sections immunostained as depicted in the panels. Arrows show the same LGALS3BP+ cells depicted in Figure 1, batches=1, organoids=3, ventricles=3. Scale bar: 30 μm in d-f and 100 μm in g-i. Abbreviations: d: days, s: sense probe, COs: cerebral organoids, NPCs: neural progenitor cells, GW: gestational week, oSVZ: outer subventricular zone.

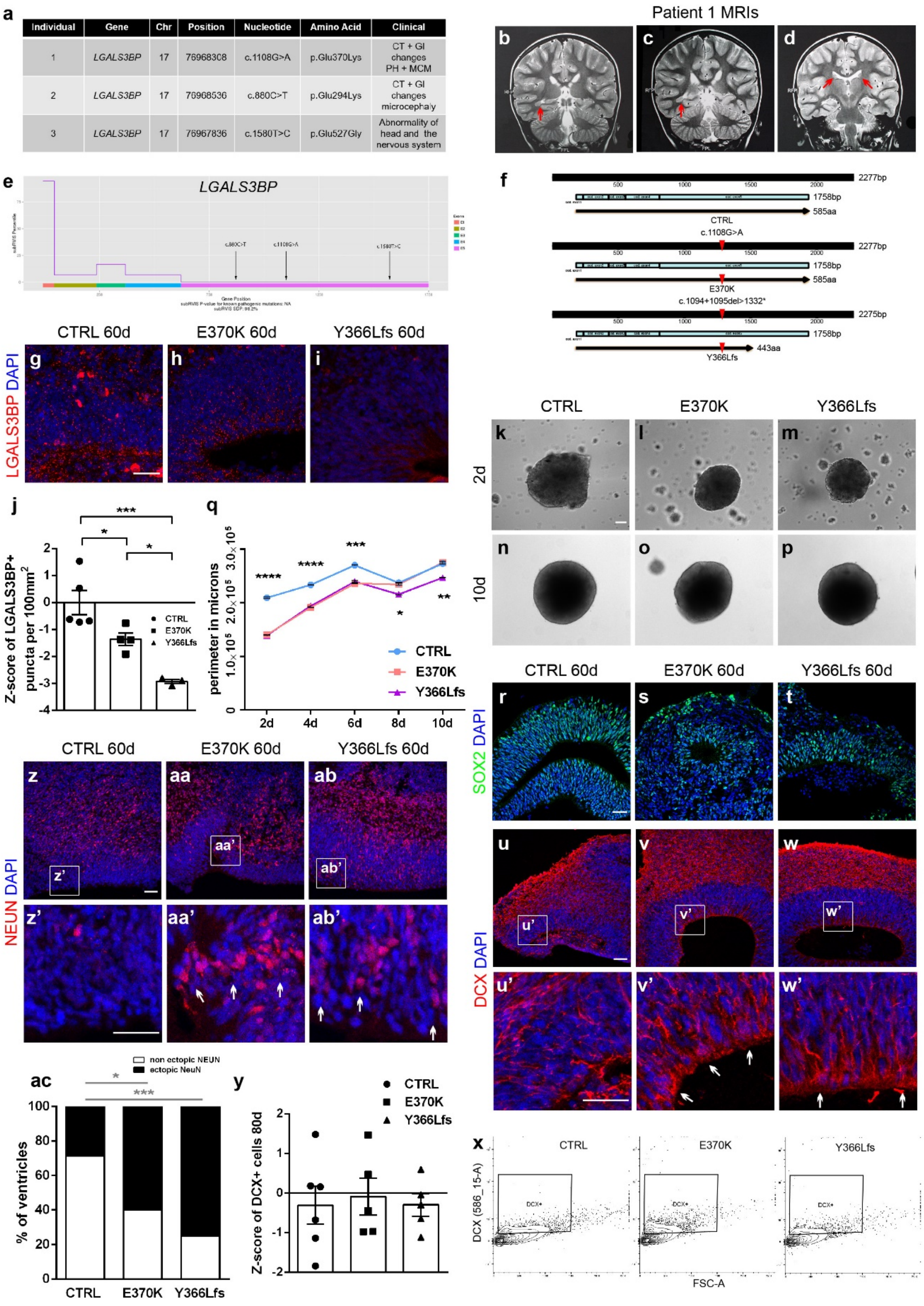

Individual	Gene	Chr	Position	Nucleotide	Amino Acid	Clinical
1	*LGALS3BP*	17	76968308	c.1108G>A	p.Glu370Lys	CT + GI changes PH + MCM
2	*LGALS3BP*	17	76968536	c.880C>T	p.Glu294Lys	CT + GI changes microcephaly
3	*LGALS3BP*	17	76967836	c.1580T>C	p.Glu527Gly	Abnormality of head and the nervous system

Supplementary Figure 2 Description. Generation of COs carrying *LGALS3BP* mutations (related to Figure 2). a, Table showing genetic information from 3 individuals with cortical malformations who have *de novo* variants in the *LGALS3BP* gene. **b-d,** Coronal cut MRI images from individual 1 demonstrating periventricular heterotopia (PH) in the right temporal horn at a point where it bends forward below the trigon and another heterotopic nodule on the contralateral peri-hippocampal region, close to a rounded and under-rotated left hippocampus (red arrows). **e,** Distribution of the three variants identified in *LGALS3BP* of this study. The x-axis represents the coding sequence of *LGALS3BP*, with each exon identified by a different color. The position of the three variants identified in the patient cohorts analyzed in this study is marked with respect to their location within this gene by arrows. The Y-axis represents the exon subRVIS score defined as a percentile across the entire coding genome as determined in[2]. Lower subRVIS percentiles correspond to more intolerant regions. **f,** Schematic representation of the two mutant iPSC lines generated using the CRISPR/Cas9 genome editing in control iPSCs. Line E370K carries a point mutation found in individual 1 located in *LGALS3BP* exon 5, while line Y366Lfs has a 2bp deletion which results in a frameshift and premature stop codon generating a truncated/KO form of *LGALS3BP* in exon 5. **g-i,** Micrographs of sections of 60d control, E370K and Y366Lfs mutant COs immunostained with LGALS3BP. **j,** Quantification of the number of puncta (LGALS3BP staining) of control, E370K and Y366Lfs mutant COs at COs culture at 60d shows that mutant COs have reduced expression of LGALS3BP. Data are shown in Z-scores, statistical analysis was based on one-way ANOVA and Turkey's multiple comparisons test *$p<0.05$, ***$p<0.001$, batches=2, organoids=3, ventricles= [5(ctrl), 4(E370K), 3(Y366Lfs)]. In j $p=0.0012$. **k-p,** Bright field micrographs of control, E370K and Y366Lfs COs showing their temporal development. **q,** Quantification of the perimeter of control, E370K and Y366Lfs mutant COs at different time points

of the COs culture shows that mutant COs have reduced size at the early stages of development. Data are shown in mean ±SEM, statistical analysis was based on two-way ANOVA and Turkey's multiple comparisons test *p<0.05, **p<0.01, ***p<0.001, ****p<0.0001, batches=2, organoids=12 per condition. **r-t,u-ab',** Micrographs of sections of 60d control, E370K and Y366Lfs mutant COs immunostained with SOX2 (r-t), DCX (u-w') or NEUN (z-ab'). Arrows depict DCX+ or NEUN+ cells located at the ventricular surface. **x,y,** FACS plots (x) and respective quantification (y) depicting the sorting of DCX+ cells in 80d control, E370K and Y366Lfs mutant Cos. Data are represented as Z-scores ±SEM. **ac,** Quantification of the percentage of ventricles having ectopic NEUN+ cells. Statistical analysis was based on one-tailed exact binomial test *p < 0.05, ***p < 0.001, batches=2, organoids=6. In ac p=0.0376 in ctrlvsE370K, p=0.0002 in ctrlvsY366Lfs. Scale bar: 100 µm in k-p and 30 µm in g-i, r-w', z-ab'. Abbreviations: CTRL: control, d: days.

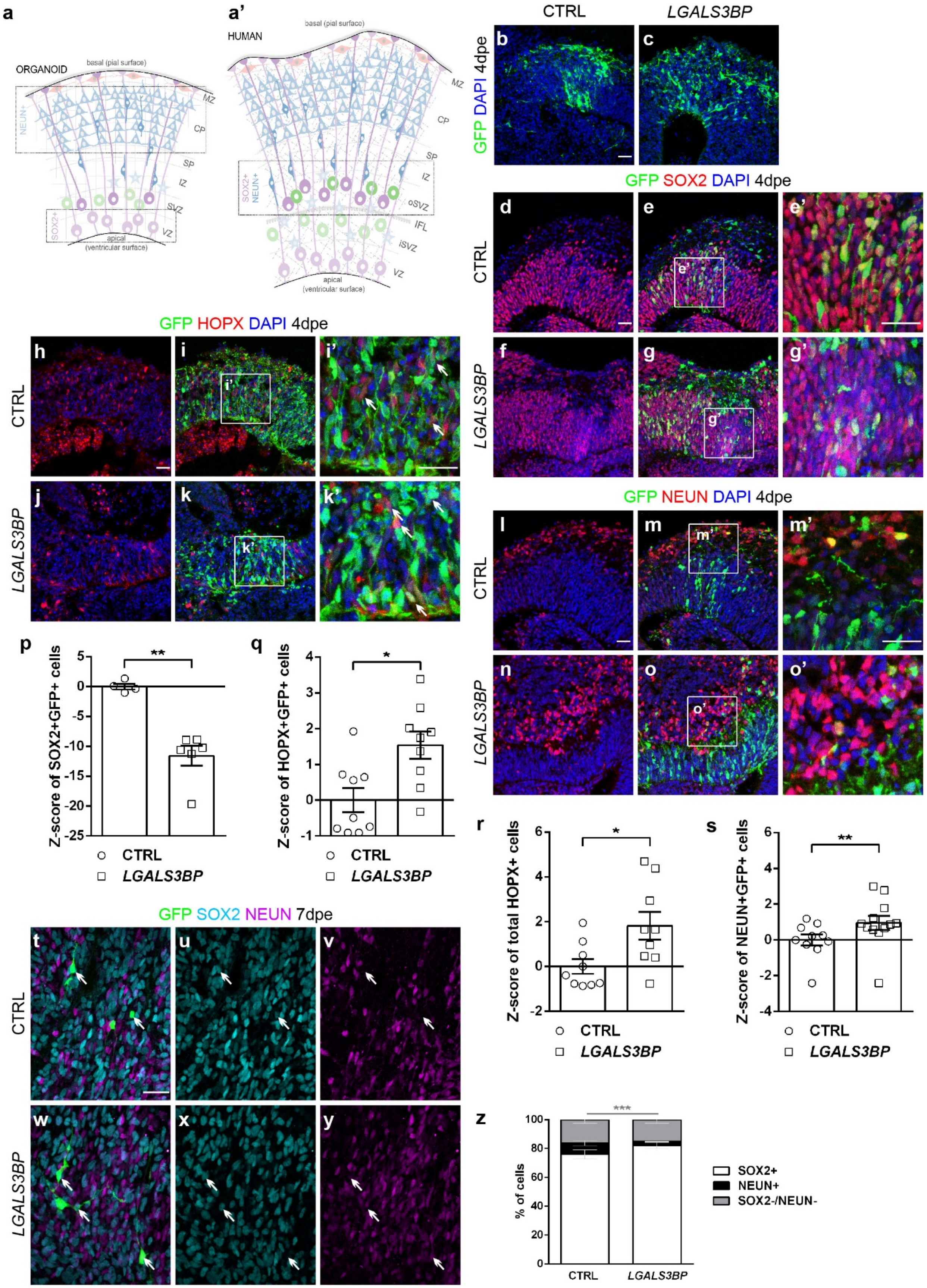
a
a'
ORGANOID
HUMAN
basal (pial surface)
apical (ventricular surface)
NEUN+
SOX2+
MZ
CP
SP
IZ
SVZ
VZ
oSVZ
IFL
iSVZ
CTRL
LGALS3BP
GFP DAPI 4dpe
GFP SOX2 DAPI 4dpe
GFP HOPX DAPI 4dpe
GFP NEUN DAPI 4dpe
GFP SOX2 NEUN 7dpe
Z-score of SOX2+GFP+ cells
Z-score of HOPX+GFP+ cells
Z-score of total HOPX+ cells
Z-score of NEUN+GFP+ cells
% of cells
SOX2+
NEUN+
SOX2-/NEUN-

Supplementary Figure 3 Description. Ectopic manipulation of *LGALS3BP* expression in COs and human fetal tissue (related to Figure 3). a,a', Schematic representation of the areas where SOX2+ and NEUN+ cells were analyzed in COs and the human fetal brain. **b,c,** Micrographs of sections of control COs immunostained with GFP after electroporation with control or *LGALS3BP* at 30d and analyzed 4 days post electroporation. **d-o',** Micrographs of sections of control COs immunostained for SOX2 (d-g'), HOPX (h-k') or NEUN (l-o') after electroporation with control or *LGALS3BP* at 40d and analyzed 4 days post electroporation. Arrows depict double-positive cells. **p,q,r,s,** Quantification of the percentage of SOX2+GFP+ (p), HOPX+GFP+ (q) to the total GFP+ population, total HOPX+ (r) or NEUN+GFP+ (s) cells upon control or *LGALS3BP* electroporation. Results are shown as Z-scores ±SEM, significance based on two-tailed Mann-Whitney U test *$p < 0.05$, **$p < 0.01$, batches=3, organoids=5, ventricles=11. In p p=0.0095 in ctrlvsLGALS3BP. In q p=0.0104 in ctrlvsLGALS3BP. In r p=0.0399 in ctrlvsLGALS3BP. In s p=0.0048 in ctrlvsLGALS3BP. **t-y,** Micrographs of organotypic slices of human fetal brains immunostained as depicted in the panels after electroporation with control or *LGALS3BP*. Analysis was performed 7 days post electroporation. Arrows depict double-positive cells. **z,** Quantification of the percentage of SOX2+GFP+, NEUN+GFP+ or SOX2-NEUN-GFP+ cells upon control or *LGALS3BP* electroporation. Results are mean ±SEM, significance based on paired t-test ***$p < 0.001$, n=4 brains in ctrl overexpression and n=3 brain in *LGALS3BP* overexpression.in z p=0.0004. Scale bar: 30 μm. Abbreviations: VZ: ventricular zone, iSVZ: inner subventricular zone, IFL: inner fiber layer, oSVZ: outer subventricular zone, IZ: intermediate zone, SP: subplate, CP: cortical plate, MZ: marginal zone, CTRL: control, dpe: days post electroporation.

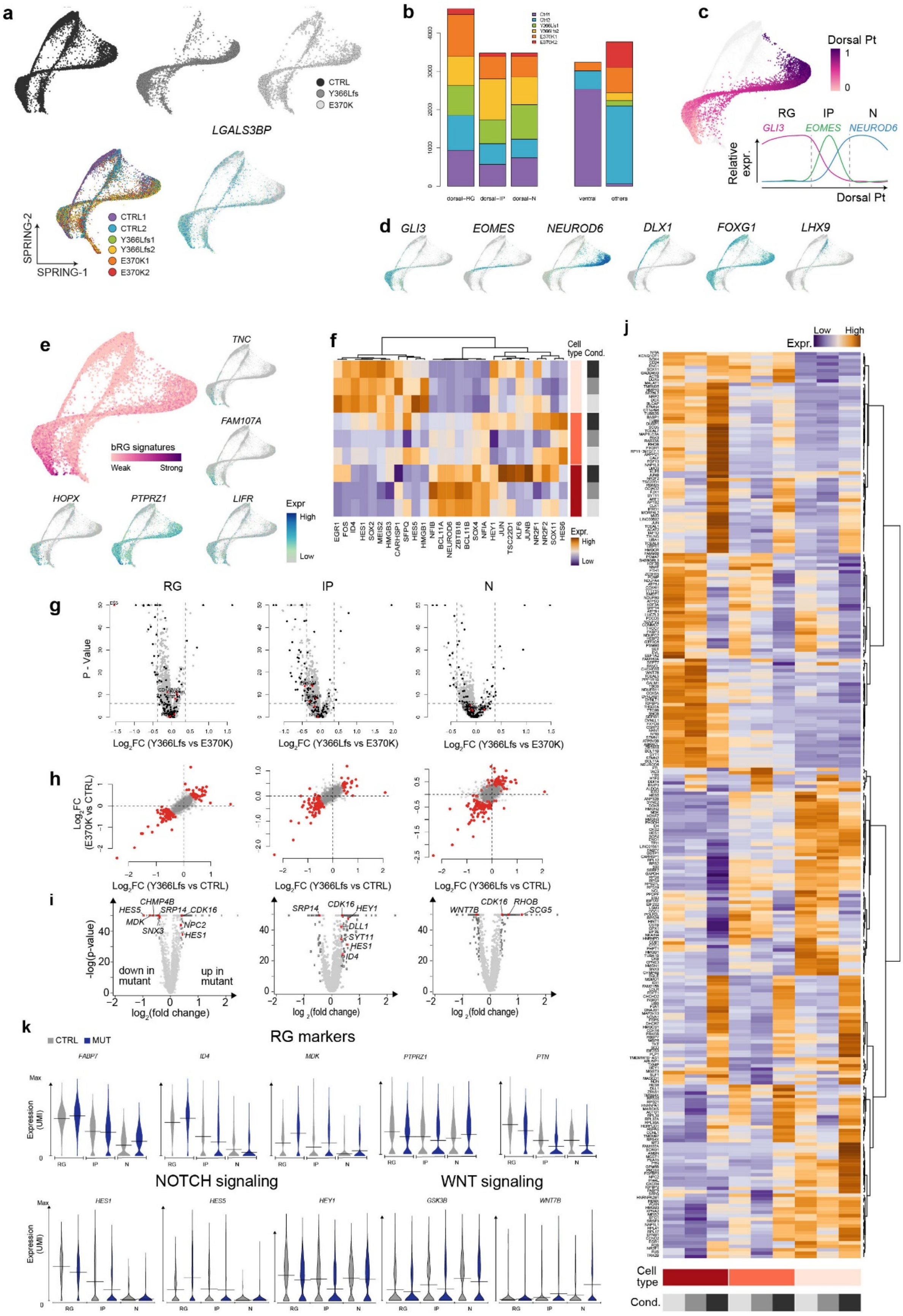
a
CTRL
Y366Lfs
E370K
LGALS3BP
CTRL1
CTRL2
Y366Lfs1
Y366Lfs2
E370K1
E370K2
SPRING-2
SPRING-1
b
dorsal-RG
dorsal-IP
dorsal-N
ventral
others
c
Dorsal Pt
RG
IP
N
GLI3
EOMES
NEUROD6
Relative expr.
d
GLI3
EOMES
NEUROD6
DLX1
FOXG1
LHX9
e
bRG signatures
Weak
Strong
TNC
FAM107A
HOPX
PTPRZ1
LIFR
Expr
High
Low
f
Cell type
Cond.
EGR1
FOS
ID4
HES1
SOX2
MEIS2
HMGB3
CARHSP1
SFPQ
HES5
HMGB1
NFIB
BCL11A
NEUROD6
ZBTB18
BCL11B
SOX4
NFIA
HEY1
JUN
TSC22D1
KLF6
JUNB
NR2F1
NR2F2
SOX11
HES6
Expr.
High
Low
g
RG
IP
N
P - Value
Log2FC (Y366Lfs vs E370K)
h
Log2FC (E370K vs CTRL)
Log2FC (Y366Lfs vs CTRL)
i
CHMP4B
HES5
SRP14
CDK16
MDK
NPC2
SNX3
HES1
-log(p-value)
down in mutant
up in mutant
log2(fold change)
SRP14
CDK16
HEY1
DLL1
SYT11
HES1
ID4
CDK16
RHOB
WNT7B
SCG5
j
Low
High
Expr.
Cell type
Cond.
k
CTRL
MUT
RG markers
FABP7
ID4
MDK
PTPRZ1
PTN
Max
Expression (UMI)
RG
IP
N
NOTCH signaling
WNT signaling
HES1
HES5
HEY1
GSK3B
WNT7B

Supplementary Figure 4 Description. scRNA sequencing analysis in *LGALS3BP* mutant COs (related to Figure 4). a, SPRING embedding of RSS integrated scRNA-seq data of COs (see Figure 4e,f) shown for cells of each condition separately (black, control; dark grey, Y366Lfs; light grey, E370K), or of each CO separately (rainbow-like color) as well as color-coded based on the expression of *LGALS3BP* (right). **b,** Bar plots show the number and proportion of each sample in each different cell type. **c,** SPRING embedding of RSS integrated CO scRNA-seq data with cells on the dorsal telencephalon trajectory color-coded in shades of magenta based on their pseudotime assignment. **d,** SPRING embedding of RSS integrated scRNA-seq data of COs color-coded based on the expression of genes marking the dorsal telencephalic radial glia (*GLI3*), intermediate progenitors (*EOMES*) and neurons (*NEUROD6*), the ventral telencephalic neurons (*DLX1*), both telencephalic trajectories (*FOXG1*) as well as the non-telencephalic trajectory (*LHX9*). **e,** Feature plots visualizing the expression of bRG marker genes (right and bottom, blue color palette) as well as a bRG signature (top left, magenta color palette) summarizing the expression of individual markers on the integrated SPRING embedding (see panels Figure 4e,f). **f,** Heatmap showing the expression profiles of genes identified as most differentially expressed between control, E370K and Y366Lfs CO cells in each of the main dorsal telencephalic cell types (radial glia, intermediate progenitors and neurons). **g,h,** Volcano (g) and scatter plots (h) illustrating the fold change of gene expression of single CO cell transcriptomes of the mutant cells (4095 E370K, 5199 Y366Lfs) among the radial glia, intermediate progenitor and neuronal populations. **i,** Volcano plots illustrating the fold change of gene expression of single CO cell transcriptomes in control (9290 cells) versus mutant cells (4095 E370K, 5199 Y366Lfs) for the dorsal telencephalon radial glia, intermediate progenitor and neuronal populations. **j,** Heatmap showing the expression of the most differentially expressed genes found in control vs mutant dorsal telencephalic cells in COs. The

sidebars on the right indicate cell type (dorsal telencephalic RG, IP, neuron in shades of red) and condition (CTRL, black; Y366Lfs, dark grey; E370K, light grey). **k,** Violin plots showing the expression distribution of genes that are up- or downregulated in mutant cells grouped in different categories: RG markers, NOTCH signaling and WNT signaling Abbreviations: CTRL: control, Pt: pseudotime, MUT: mutant, RG: radial glial, bRG: basal radial glial, IP: intermediate progenitor, N: neuron, Cond.: condition, Expr.: expression, UMI: unique molecular identifiers, M.

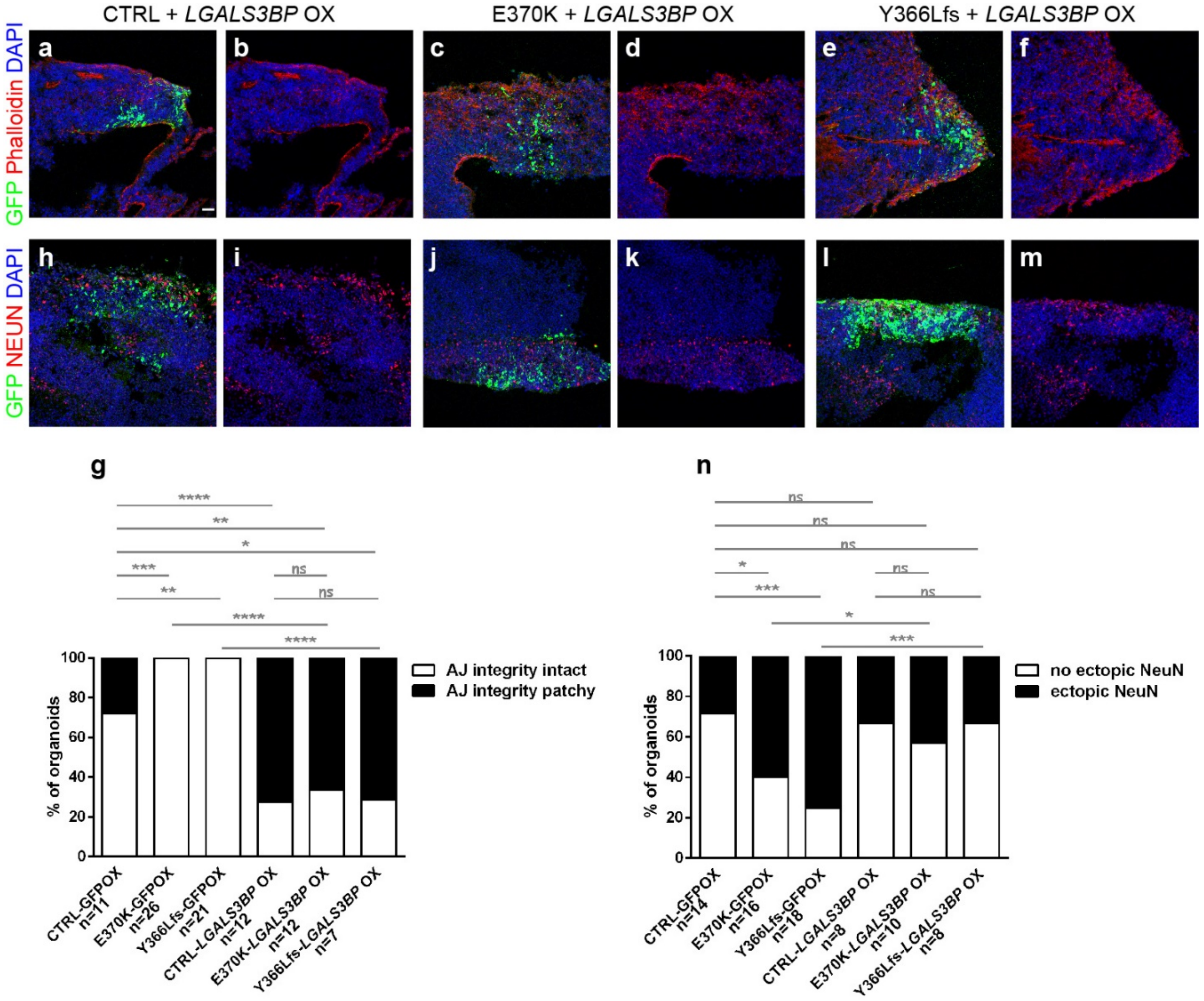

Supplementary Figure 5 Description. Overexpression of *LGALS3BP* in mutant COs can rescue the phenotype (related to Figure 5). a-f,h-m, Micrographs of sections of control and mutant COs immunostained as depicted in the panels after electroporation of *LGALS3BP* at 40d and analysed 4 days post electroporation. **g,n,** Quantification of the percentage of COs with intact of patchy apical junction (g) or with ectopic neurons (n) upon overexpression of *LGALS3BP* in control, E370K or Y366Lfs mutant COs. Data are shown in percentages, statistical significance was based on one-tailed exact binomial test *p<0.05, **p<0.01, ***p<0.001, ****p < 0.0001, batches=1, organoids=6, ventricles=10. In g p=0.0057 in ctrGFPvsE370K-LGALS3BP OX, p=0.0206 in ctrGFPvsY366Lfs-LGALS3BP OX, p=0.0003 in ctrlGFPvsE370KGFP OX,

p=0.0022 in ctrlGFPvsY366LfsGFP OX. In n p=0.0376 in ctrlGFPvsE370KGFP OX, p=0.0002 in ctrlGFPvsY366LfsGFP OX, p=0.0008 in Y366LfsGFPvsY366LfsLGALS3BP OX. Scale bar: 30 μm. Abbreviations: d: days, CTRL: control, OX: overexpression, %: percentage.

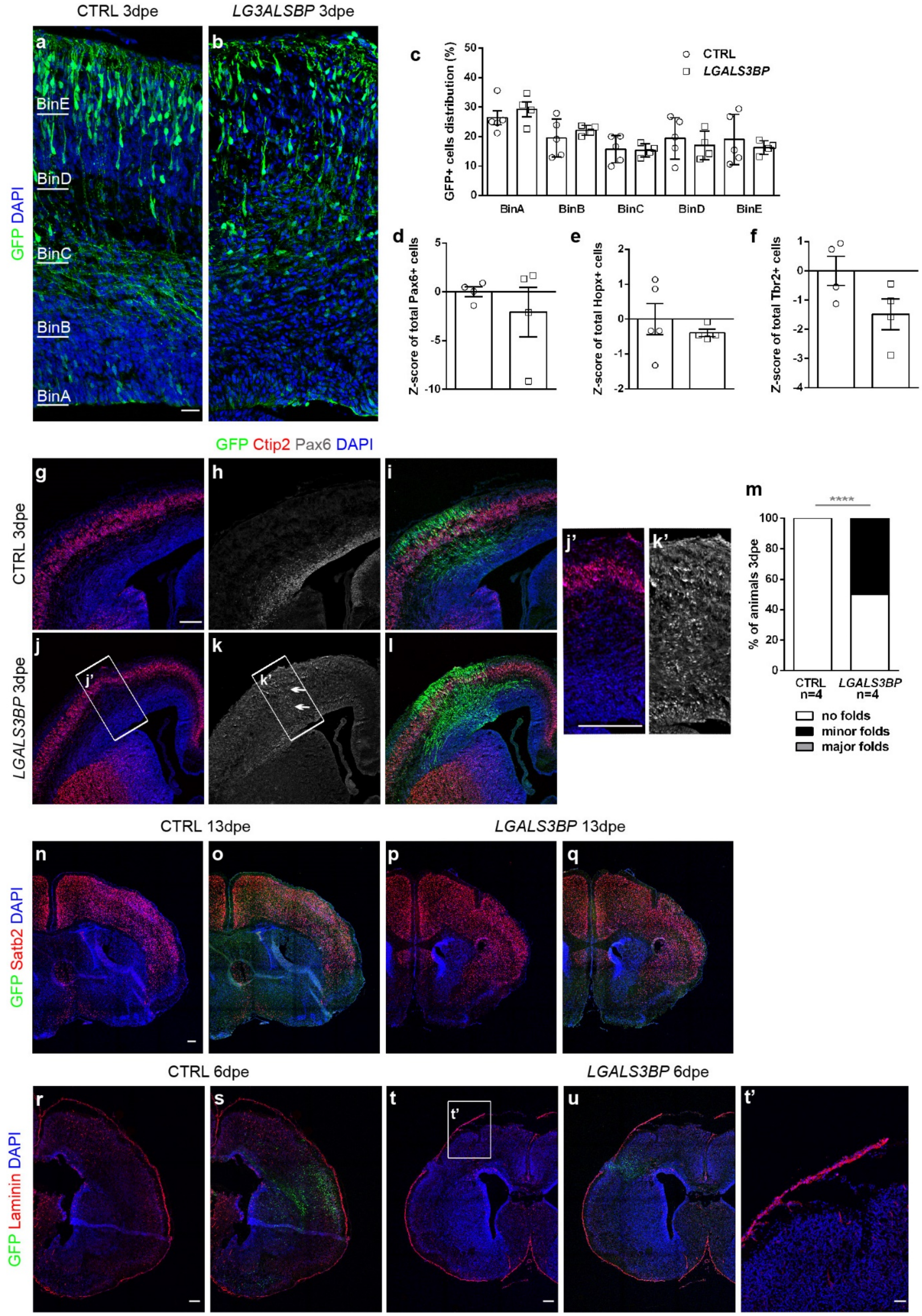
CTRL 3dpe
LG3ALSBP 3dpe
GFP DAPI
BinE
BinD
BinC
BinB
BinA
CTRL
LGALS3BP
GFP+ cells distribution (%)
Z-score of total Pax6+ cells
Z-score of total Hopx+ cells
Z-score of total Tbr2+ cells
GFP Ctip2 Pax6 DAPI
CTRL 3dpe
LGALS3BP 3dpe
% of animals 3dpe
****
CTRL n=4
LGALS3BP n=4
no folds
minor folds
major folds
CTRL 13dpe
LGALS3BP 13dpe
GFP Satb2 DAPI
CTRL 6dpe
LGALS3BP 6dpe
GFP Laminin DAPI

Supplementary Figure 6 Description. Overexpression of *LGALS3BP* in mice results in the formation of an oSVZ-like zone (related to Figure 6). a,b, Micrographs of sections of mice immunostained with GFP after electroporation of control or *LGALS3BP* at embryonic day 13 and analyzed 3 days post electroporation. **c,** Quantification of the distribution of the GFP+ cells in the developing cortex. Data are shown as mean ±SEM, n=6 ctrl, n=5 LGALS3BP mouse brains. **d,e,f,** Quantification of the total number of Pax6+ (d), Hopx+ (e), or Tbr2+ cells (f). Data are shown as Z-scores ±SEM n=5 ctrl, n=4 LGALS3BP mouse brains. **g-l,** Micrographs of sections of mice immunostained for Ctip2 (red) and Pax6 (grey) after electroporation of control or *LGALS3BP* at embryonic day 13 and analyzed 3 days post electroporation. Arrows and white boxes indicate the region in the overexpressing mice which shows the initiation of fold-like structure in the neuronal layers and the existence of Pax6+ basally located progenitors. **m,** Quantification of the percentage of animals that had no folds (white) or minor folds (black). Data are shown as percentages. Statistical significance was based on one-tailed exact binomial test **** $p<0.0001$, n=4. **n-t',** Micrographs of sections of mice immunostained for Satb2 after electroporation of control or *LGALS3BP* at embryonic day 13 and analyzed 13 days post electroporation (n-q) or with Laminin after electroporation of control or *LGALS3BP* at embryonic day 13 and analyzed 6 days post electroporation, n=3ctrl and n=7 *LGALS3BP* overexpressed animals. Mice overexpressing *LGALS3BP* have fold-like structures and a proper basal lamina (laminin). Scale bar: 30 µm in a,b, 200 µm in g-l,n-u, 50 µm in t'. Abbreviations: CTRL: control, dpe: days post electroporation, %: percentage.

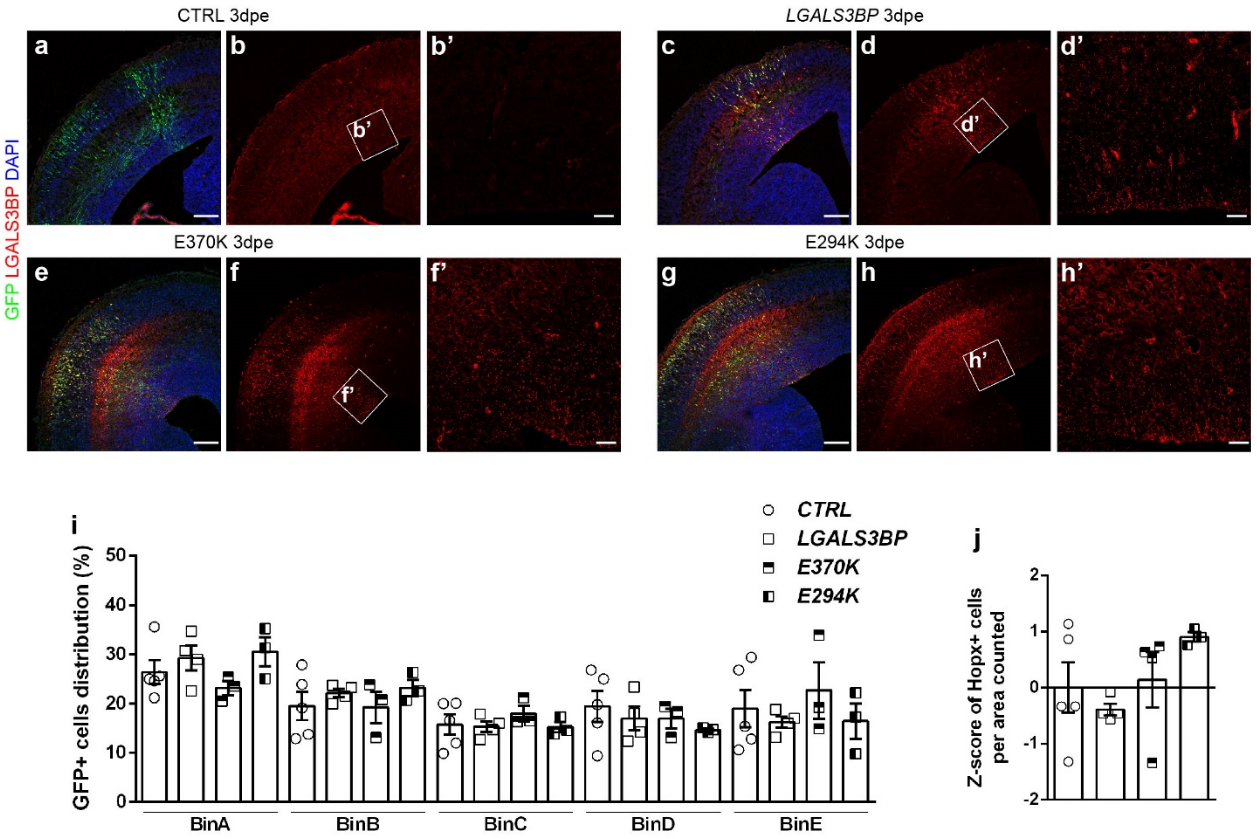

Supplementary Figure 7 Description. Overexpression of mutant forms of *LGALS3BP* in mice does not affect the distribution of cells (related to Figure 7). a-h', Micrographs of sections of mice immunostained as depicted in the panels after electroporation of CTRL plasmid or wt, E370K or E294K *LGALS3BP* at embryonic day 13 and analyzed 3 days post electroporation. **i,j,** Quantification of the distribution of the GFP+ cells in the developing cortex (i) and of the total number of Hopx+ (j) cells. Data are shown as mean ±SEM (i) or as Z-scores (j), n= 5 in ctrl, 4 in LGALS3BP, 3in E370K, 3in E294K. Scale bar: 200 μm. Abbreviations: CTRL: control, dpe: days post electroporation.

a

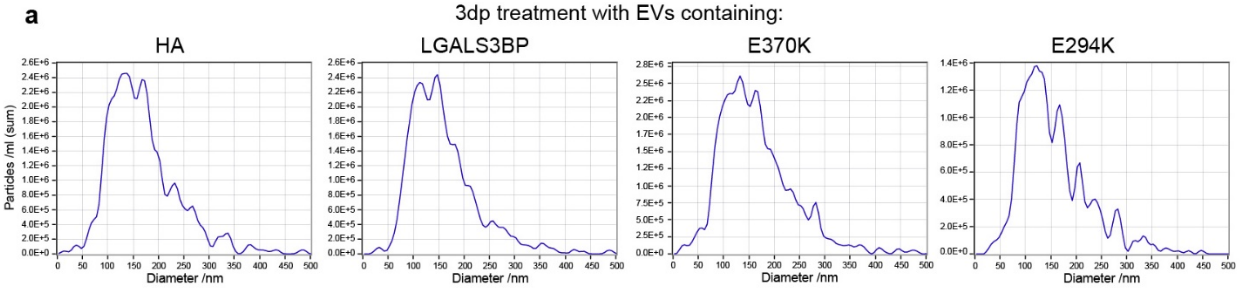

b

Cellular phenotype	Human OX	Human MUT	COs OX	COs MUT	Mice OX
Cortical thickness changes	-	+	-	-	-
Gyrification changes	-	+	Not assessed	Not assessed	+
Ectopic neurons	-	+	-	+	-
Changes in the number of NPCs	-	Not assessed	+	-	-
Changes in the position of NPCs	More basal locations	Not assessed	More basal locations	More apical locations	More basal locations
Changes in the morphology of NPCs	-	Not assessed	+	+	-
Changes in the apical belt	Not assessed	Not assessed	Loss of integrity	ticker	Loss of integrity
Cell non-autonomous phenotype	Not assessed	Not assessed	+	Not assessed	+
EVs mediated phenotype	Not assessed	Not assessed	Not assessed	+	+

Supplementary Figure 8 Description. Size of LGALS3BP containing EVs (related to Figure 8). a, Plots showing the number and size of EVs isolated from SH-SY5Y cells following electroporation with HA, HA-LGALS3BP, HA-E370K or HA-E294K. **b, a** Summary table showing the phenotypes observed in human feral cortexes, in COs and mice following manipulation of LGALS3BP Abbreviations: OX: overexpression, MUT: mutant, NPCs: neural progenitor cells, EVs: extracellular vesicles.

References

1. Nowakowski, T. J. *et al.* Spatiotemporal gene expression trajectories reveal developmental hierarchies of the human cortex. *Science* **358**, 1318–1323 (2017).
2. Gussow, A. B., Petrovski, S., Wang, Q., Allen, A. S. & Goldstein, D. B. The intolerance to functional genetic variation of protein domains predicts the localization of pathogenic mutations within genes. *Genome Biology* **17**, 9 (2016).
