# Supplementary material for: Extracellular LGALS3BP regulates neural progenitor position and relates to human cortical complexity
Source: Nat Commun. 2021 Nov 2;12:6298. doi: 10.1038/s41467-021-26447-w (PMC8564519; doi:10.1038/s41467-021-26447-w)
Supplement: Supplementary file 3 — Reporting summary. [file 41467_2021_26447_MOESM3_ESM.pdf]

nature portfolio

Corresponding author(s): Silvia Cappello, Stephen P. Robertson

Last updated by author(s): Aug 31, 2021

Reporting Summary

Nature Portfolio wishes to improve the reproducibility of the work that we publish. This form provides structure for consistency and transparency in reporting. For further information on Nature Portfolio policies, see our Editorial Policies and the Editorial Policy Checklist.

Statistics

For all statistical analyses, confirm that the following items are present in the figure legend, table legend, main text, or Methods section.

n/a	Confirmed	
☐	☒	The exact sample size (*n*) for each experimental group/condition, given as a discrete number and unit of measurement
☐	☒	A statement on whether measurements were taken from distinct samples or whether the same sample was measured repeatedly
☐	☒	The statistical test(s) used AND whether they are one- or two-sided<br>*Only common tests should be described solely by name; describe more complex techniques in the Methods section.*
☒	☐	A description of all covariates tested
☐	☒	A description of any assumptions or corrections, such as tests of normality and adjustment for multiple comparisons
☐	☒	A full description of the statistical parameters including central tendency (e.g. means) or other basic estimates (e.g. regression coefficient) AND variation (e.g. standard deviation) or associated estimates of uncertainty (e.g. confidence intervals)
☐	☐	For null hypothesis testing, the test statistic (e.g. *F*, *t*, *r*) with confidence intervals, effect sizes, degrees of freedom and *P* value noted<br>*Give P values as exact values whenever suitable.*
☒	☐	For Bayesian analysis, information on the choice of priors and Markov chain Monte Carlo settings
☒	☐	For hierarchical and complex designs, identification of the appropriate level for tests and full reporting of outcomes
☐	☒	Estimates of effect sizes (e.g. Cohen's *d*, Pearson's *r*), indicating how they were calculated

Our web collection on statistics for biologists contains articles on many of the points above.

Software and code

Policy information about availability of computer code

Data collection	Leica software Las X, Folw Jo VX, Image lab 6.0.1, snap gene 1.1.3, ZetaView Software 8.05.12 SP1, Genomics Cell Ranger 2.0 software, Xcalibur software version 3.0sp2, MaxQuant software 1.6.2.10, Perseus 1.5.1.6
Data analysis	Image J win64, GraphPad Prism 6, Illustrator CC, Photoshop CS5

For manuscripts utilizing custom algorithms or software that are central to the research but not yet described in published literature, software must be made available to editors and reviewers. We strongly encourage code deposition in a community repository (e.g. GitHub). See the Nature Portfolio guidelines for submitting code & software for further information.

Data

Policy information about availability of data

All manuscripts must include a data availability statement. This statement should provide the following information, where applicable:

- Accession codes, unique identifiers, or web links for publicly available datasets
- A description of any restrictions on data availability
- For clinical datasets or third party data, please ensure that the statement adheres to our policy

All data supporting the findings described in this manuscript are available in the article and in the Supplementary Information, and from the corresponding author upon reasonable request.
Data illustrated in Extended Data Figure 1a are obtained from doi: 10.1126/science.aap8809 and sited in this manuscript.
The scRNA-seq data used in this study have been deposited in the ArrayExpress under accession number E-MTAB-10485 [https://www.ebi.ac.uk/arrayexpress/experiments/E-MTAB-10485/].
The mass spectrometry proteomics data and mass spectrometry secretome data have been deposited to the ProteomeXchange Consortium via the PRIDE[86]

partner repository with the dataset identifier PXD015878.
Source data are provided with this paper (Supplementary Table 6).
Web resourches used in this manuscript are listed here:
DECIPHER, https://decipher.sanger.ac.uk/
scRNA sequencing data from human fetal cortex https://cells.ucsc.edu/?ds=cortex-dev
gnomAD Browser, v.r2.0.2, http://gnomad.broadinstitute.org/

Field-specific reporting

Please select the one below that is the best fit for your research. If you are not sure, read the appropriate sections before making your selection.

☒ Life sciences ☐ Behavioural & social sciences ☐ Ecological, evolutionary & environmental sciences

For a reference copy of the document with all sections, see nature.com/documents/nr-reporting-summary-flat.pdf

Life sciences study design

All studies must disclose on these points even when the disclosure is negative.

Sample size	For mice the minimum number was selected according to the 3R (Replacement, Reduction and Refinement) as previously (e.g. Cappello et al 2013 Nat.Gen.)<br>For organoids we applied the same sample size determination by following previous publications (e.g. Klaus et al 2019 Nat.Med.)
Data exclusions	No data were excluded from the analysis
Replication	All experiments were reproduced at least 3 times independently. All attempts for replications were successful.
Randomization	We do not have experimental groups, only 2 patients were available. For all the other data we did not perform any randomization
Blinding	The majority of the data are acquired in a blinded manner. Some of the data were calculated from more than one person.

Reporting for specific materials, systems and methods

We require information from authors about some types of materials, experimental systems and methods used in many studies. Here, indicate whether each material, system or method listed is relevant to your study. If you are not sure if a list item applies to your research, read the appropriate section before selecting a response.

Materials & experimental systems

n/a	Involved in the study	
☐	☐	Antibodies
☐	☒	Eukaryotic cell lines
☒	☐	Palaeontology and archaeology
☐	☒	Animals and other organisms
☐	☒	Human research participants
☒	☐	Clinical data
☒	☐	Dual use research of concern

Methods

n/a	Involved in the study	
☒	☐	ChIP-seq
☐	☒	Flow cytometry
☐	☒	MRI-based neuroimaging

Antibodies

Antibodies used

All antibodies are commercially available antibodies commonly used is several other publications. Every antibody was then validated in the lab to exclude non-specific signals due to secondary antibodies. A full list of the antibodies used is found in the manuscript and here:
Antibodies
Antigen Dilution Vendor Catalog # Lot #
PAX6 1:500 Biolegend PRB-278p B244513
LGALS3BP 1:100 eBioscience BMS146 -
SOX2 1:500 Cell Signalling 27485 2
HOPX 1:1000 Sigma Aldrich HPA030180 B105571
CTIP2 1:500 Abcam ab18465 GR322373-4
SATB2 1:500 Abcam Ab51502 GR2075794
TBR1 1:500 Abcam ab31940 GR3217067-1
LAMININ 1:500 Millipore AB2034 2558444
MAP2 1:500 Sigma Aldrich M4403 035MN4780V

PALS1 1:500 Sigma Aldrich 07-708 H0907
β-CATENIN 1:500 BD Biosciences 610154 76645
PH3 1:500 Millipore 06-570 3113883
ARL13B 1:200 Proteintech 17711-1-AP -
GAPDH 1:6000 Millipore CB1001 2896484
GFP 1:1000 Aves Lab GFP-1020 697986
NESTIN 1:200 Millipore MAB5326 3112610
PAN-CADHERIN Sigma Aldrich C1821 064M4764
CD82 1:250 Santa cruz sc-1087 J2814
TBR2 1:500 Abcam ab23345 GR33045451
PHALLOIDIN (ACTIN) 1:40 Thermo Fisher A12381 1743642
DoubleCortin (DCX) 1:2000 Millipore AB2253 2787730
NEUN 1:500 Millipore MAB377 2742283
KI67 1:500 DAKO M7248 20017551
FABP7 1:1000 Millipore ABN14 3160120
HA 1:1000 Santa cruz Sc-7392 K1918
CD81 1:250 Santa cruz Sc-166029 D0419
Alexa Fluor® 647 Goat Anti-Mouse IgG (H+L) 1:1000 Life-Technologies A-21235 -
Alexa Fluor® 647 Goat Anti-Rabbit IgG (H+L) Antibody 1:1000 Life-Technologies A-21244 2086730
Alexa Fluor® 647 Goat Anti-Guinea Pig IgG (H+L), highly cross-adsorbed 1:1000 Life-Technologies A-21450 2026140
Alexa Fluor® 546 Goat Anti-Mouse IgG1 (γ1) 1:1000 Life-Technologies A- 21123 1722393
Alexa Fluor® 546 Goat Anti-Mouse IgG (H+L) 1:1000 Life-Technologies A-11003 -
Alexa Fluor® 546 Goat Anti-Rabbit IgG (H+L) 1:1000 Life-Technologies A-11010 1971417
Alexa Fluor® 546 Goat Anti-Rat IgG (H+L) 1:1000 Life-Technologies A-11081 -
Alexa Fluor® 488 Goat Anti-Chicken IgG (H+L) Antibody 1:1000 Life-Technologies A-11039 2079383

Validation

All antibodies that are commercially available were used according to manufactures instructions. Every antibody was validated in the lab to exclude non-specific signals due to secondary antibodies using as positive controls control sections (in the case of immunofluorescence) and blots (in the case of western blots) available in the lab.

Eukaryotic cell lines

Policy information about cell lines

Cell line source(s)

Commercially available human cancer cell lines (SH-SY5Y) were used in this study (cat number CRL-226 from ATCC). Commercially available control fibroblasts
which were reprogrammed in the lab to iPSCs were included in this study (ATCC)

Authentication

The control iPSC line used in this study was genotyped (Klaus et al Nat. Med. 2019). The genome edited lines generated for the purposes of this study were genotyped via sequencing of the region of interest and the data are available in the manuscript. The SH-SY5Y cells were not authenticated because they were bought from ATCC and authenticated from the company

Mycoplasma contamination

All cells were tested for mycoplasma monthly. All cell used were tested negative.

Commonly misidentified lines
(See ICLAC register)

no commonly misidentified lines were used in this study

Animals and other organisms

Policy information about studies involving animals; ARRIVE guidelines recommended for reporting animal research

Laboratory animals

Female C57BL/6 mice from 2 to 6 months of age were used in this study. Housing conditions are included in the manuscript

Wild animals

no wild animals were used in the study

Field-collected samples

no field collected samples were used in the study

Ethics oversight

Animal experiments were approved by the Government of Upper Bavaria under license number 55.2-1-54-2532-79-2016

Note that full information on the approval of the study protocol must also be provided in the manuscript.

Human research participants

Policy information about studies involving human research participants

Population characteristics

Two research participants are described. Case 1 is male and aged 3 years. Case 2 is female and aged 1 year. There is no parental consanguinity.
Human embryonic tissues form four different embryos at gestational week (GW) 14-18 containing prefrontal cortex from one hemisphere were used

	The iPSCs used here were derived from fibroblasts of male (CTRL: newborn). Mutant iPSCs were generated form the control iPSC line.
Recruitment	Participants were recruited via two different protocols. Case 1 was ascertained via physician initiated referral to a research programme dedicated to defining the genetic causes of periventricular nodular heterotopia. No exclusions were imposed for recruitment into this protocol apart from prior screening FLNA for pathogenic variants.<br><br>Case 2 was ascertained through the Deciphering Developmental Disorders study. The study recruits participants with developmental conditions via National Health Service provisioned genetic clinics. Most participants in this study were pre-screening by chromosomal microarray analysis. Samples from these individuals were subjected to exome sequencing. Variants of uncertain significance are lodged on the Decipher database. This patient was recruited to this study through identification of an LGAL3BP variant registered on this database.<br><br>Protocols for ascertainment of these two research subjects therefore hold minimal potential for recruitment bias.
Ethics oversight	The first patient was consented to participate under the University of Otago consent protocol. Parents or legally authorised representatives gave signed consent to participate in this study under protocols that were approved either from the Southern regional Ethics Committee O03/016 and the New Zealand Ethics Committee MEC08/08/094 subjects gave consent for the generation of iPS cells. For the New Zealand based study general sharing of individual exome sequences was not approved on confidentiality grounds. The second patient, of whom clinical data were obtained, was identified within the Deciphering Developmental Disorders (DDD) study [45]. This study has UK Research Ethics Committee approval (10/H0305/83), granted by the Cambridge South Research Ethics Committee and was conducted in accordance to the criteria set by the Declaration of Helsinki. All patients or their guardians provided written informed consent.

Note that full information on the approval of the study protocol must also be provided in the manuscript.

Flow Cytometry

Plots

Confirm that:

- [x] The axis labels state the marker and fluorochrome used (e.g. CD4-FITC).
- [x] The axis scales are clearly visible. Include numbers along axes only for bottom left plot of group (a 'group' is an analysis of identical markers).
- [x] All plots are contour plots with outliers or pseudocolor plots.
- [x] A numerical value for number of cells or percentage (with statistics) is provided.

Methodology

Sample preparation	Samples were collected form at least 6 organoids generated in at least 2 independent batches. Full experimental description of the sample preparation is included in the materials and methods section of the manuscript.
Instrument	FACS analysis was performed at a FACS Aria (BD) in BD FACS Flow TM medium, with a nozzle diameter of 100 µm
Software	Flow Jo
Cell population abundance	All the samples analyzed by flow cytometry were cells dissociated from cerebral organoids from patients and controls and stained with different antibodies. For each run, 10000 cells were analyzed based on previous experience. Gating strategy SSC-A/FSC-A gates were done to exclude cell debris and FSC-W/FSC-A to collect single cells. The boundary between positive and negative staining cells was set according to the isotype control for each of the antibody used in this project.
Gating strategy	Debris and aggregated cells were gated out by forward scatter, sideward scatter; single cells were gated out by FSC-W/FSC-A. Gating for fluorophores was done using samples stained with secondary antibody only. Flow rate was below 500 events/sec

- [x] Tick this box to confirm that a figure exemplifying the gating strategy is provided in the Supplementary Information.

Magnetic resonance imaging

Experimental design

Design type	Regular diagnostic, structural MRI imaging at 3T on a sedated patient.
Design specifications	MRI analysis was conducted on data acquired in a clinical setting and processed using a gold-standard software pipeline, no further design specifications are applicable.
Behavioral performance measures	Behavioral performance was not measured in our study because our hypothesis was only focused on correlating the gene variation with developmental abnormalities of the human cerebral cortex.

Acquisition

Imaging type(s)	Structural
Field strength	3T
Sequence & imaging parameters	Gradient echo, spin echo, inversion recovery
Area of acquisition	Whole brain analysis was used for brain imaging acquired with T1-weighted sequences, no diffusion MRI was used.

Diffusion MRI ☐ Used ☒ Not used

Preprocessing

Preprocessing software	MRI acquisition and reconstruction: GE 15, LX, MR Software release: 15.0_M4_0910.a<br>MRI segmentation and morphometric evaluation: FreeSurfer 5.3.0
Normalization	Surface-based registration of single subjects on template
Normalization template	Surface-based template obtained by averaging 8 age-matched controls
Noise and artifact removal	Noise/artifact removal algorithms were applied by the acquisition software embedded in the MRI scanner.
Volume censoring	Volume censoring was not applied to the preprocessing pipeline

Statistical modeling & inference

Model type and settings	GLM model - two-tailed paired t-test
Effect(s) tested	Effect of gene mutation on morphometric features (cortical thickness and local gyrification index)

Specify type of analysis: ☒ Whole brain ☐ ROI-based ☐ Both

Statistic type for inference (See Eklund et al. 2016)	cluster-wise correction - 1000 iterations, z=1.3, p<0.05
Correction	Monte Carlo approach

Models & analysis

n/a	Involved in the study
☒	☐ Functional and/or effective connectivity
☒	☐ Graph analysis
☒	☐ Multivariate modeling or predictive analysis
