# Supplementary material for: Extracellular LGALS3BP regulates neural progenitor position and relates to human cortical complexity
Source: Nat Commun. 2021 Nov 2;12:6298. doi: 10.1038/s41467-021-26447-w (PMC8564519; doi:10.1038/s41467-021-26447-w)
Supplement: Supplementary file 4 — Description of Additional Supplementary Files. [file 41467_2021_26447_MOESM4_ESM.pdf]

Description of Additional Supplementary Files

Supplementary Data 1: LGALS3BP organoids_Differentially expressed genes. Table 1includs quality control metrics, detected genes/transcripts their expression values as well as cell number for each cluster as shown in the barplot in Fig 4h. and genes and their respective expression in each of the clusters as shown in the heatmap in Extended data Fig. 4e.

Supplementary Data 2: LGALS3BP organoids_Differentially expressed proteins in the proteome. Table 2 includes detected proteins and their expression values in the whole proteome from organoid cells.

Supplementary Data 3: LGALS3BP organoids_Differentially expressed proteins in the secretome. Table 3 includes detected proteins and their expression values in secretome from the organoid medium.

Supplementary Data 4: LGALS3BP_crisprcas9 editing lines info. Table 4 includes information regarding the design of LGALS3BP guides and off-targets for generating the mutant iPSC lines as well as the final sequence of the alleles after the genomic editing.

Supplementary Data 5: List of primers. Table 5 includes the sequences of all the primers used for iPSC colonies screening and the generation of the plasmids expressing the mutant variants of LGALS3BP.

Supplementary Data 6: Source data for graphical representations and statistical descriptions. Table 6 includes raw data and information regarding the statistical analysis that was applied in each graph of this manuscript.
